# Patient-Centered Radiology with FHIR: an Introduction to the Use of FHIR to Offer Radiology a Clinically Integrated Platform

**DOI:** 10.1007/s10278-018-0087-6

**Published:** 2018-05-03

**Authors:** Peter I. Kamel, Paul G. Nagy

**Affiliations:** Department of Radiology, Johns Hopkins Medicine, 601 N Caroline St, Baltimore, MD 21287 USA

**Keywords:** FHIR, HL7, Hackathon, Integration, Patient-centered radiology

## Abstract

Fast Healthcare Interoperability Resources (FHIR) is an open interoperability standard that allows external software to quickly search for and access clinical information from the electronic medical record (EMR) in a method that is developer-friendly, using current internet technology standards. In this article, we highlight the new FHIR standard and illustrate how FHIR can be used to offer the field of radiology a more clinically integrated and patient-centered system, opening the EMR to external radiology software in ways unfeasible with traditional standards. We explain how to construct FHIR queries relevant to medical imaging using the Society for Imaging Informatics in Medicine (SIIM) Hackathon application programming interface (API), provide sample queries for use, and suggest solutions to offer a patient-centered, rather than an image-centered, workflow that remains clinically relevant.

## Introduction

The field of radiology has been increasingly challenged by balancing multiple software platforms which often separate the picture archiving and communication system (PACS) from the electronic medical record (EMR). In a national survey conducted in 2016, more than half of academic hospitals reported no integration between the PACS and the EMR [1]. Another study concluded there is “an alarming lack of communication of pertinent medical information to the radiologist,” requiring the radiologist to be able to access the EMR since awareness of important clinical information demonstrates a clear impact on the quality of radiologic interpretation [2]. For certain diagnostic studies, as low as 34% of the time would a radiologist review the EMR for clinical information [3].

The typical radiology interpretation process often starts with viewing images without reviewing supporting clinical information to avoid framing bias [4]. After initial review, depending on recognition of the need for additional information, will the radiologist search into the EMR for the patient’s history of present illness, medical history, or relevant laboratory work. These explorations into the medical record often require opening and authenticating with additional software, querying for and selecting the patient, and clicking and scrolling to identify the relevant note and find relevant lab work. Not only can this be time-consuming for radiologists, but information is presented in fragmented pieces. Important information may be missed or information may be taken out of context. One example is noting an elevated leukocyte count and using it to drive an infectious diagnosis, when the patient could have a documented noninfectious increase in white cells from a medication such as corticosteroids.

Radiologists having access to relevant information in the EMR, can improve value of patient care [2]. With the advent of new application program interfaces (APIs), typical barriers for external software to integrate with clinical systems have been greatly reduced. Tools like DICOMWeb, the Society for Imaging Informatics in Medicine (SIIM) Workflow Initiative in Medicine (SWIM), and Fast Healthcare Interoperability Resources (FHIR) are a variety of upcoming APIs that encourage developer integration of medical images, workflow data, and clinical information respectively. This paper will explore the new FHIR standard and demonstrate how it can integrate relevant EMR data into the clinical workflow of a radiologist.

### FHIR

FHIR is an interoperability standard developed by Health Level Seven (HL7) that functions as an API for developers to access needed clinical information from the EMR. FHIR leverages decades of experience in healthcare interoperability with HL7 Version 2, the predominant interoperability standard used today in medical systems for EMR integration. HL7 Version 2 utilized a push-based broadcast messaging system to pass information between systems. To access information with HL7 Version 2, external software would need to listen to a stream of messages that describe clinical events. Only upon receipt of a message could external software collect and process the information. This message-based system of HL7 differs from FHIR’s service-oriented system which permits external software to ask specific clinical questions and obtain an immediate response. An example is being able to ask, “Who are the patients that are currently admitted to the hospital?” with FHIR, instead of compiling a list of patients by listening to a stream of admission and discharge events with HL7 messages. This service-based approach promises benefits such as interoperability, extensibility, and speed when searching for specific information in many different clinical applications [5–7].

A second key advantage of FHIR is the World Wide Web Consortium (W3C) compliant format that follows a language and structure well-established and commonly utilized in the web development community. It is the same format that organizations such as Facebook or Twitter use in their APIs. The structure is called a representational state transfer (RESTful) architecture which standardizes methods to search for, update, and delete data. This also differs from HL7 which required creation of its own transport mechanism when first developed in the 1980s.

One clear benefit with FHIR for the field of radiology is the ability to quickly probe for relevant clinical information. The power of FHIR comes from the simplicity of the queries to fetch specific data, eliminating the need for complex searches in the EMR. One example is the ability to query specifically for the presence of an iodinated contrast allergy, instead of authenticating with the EMR and searching through a list of all the patient’s allergies. With FHIR, imaging software can query clinical information mined from the EMR and integrate it with other clinical systems used by the radiologist.

## Review

### Getting Up and Running

SIIM hosts a cloud-based FHIR server as part of its annual hackathon [8]. This server is accessible to SIIM members yearlong after registration for an api key. The URL to the FHIR server is:

- http://api.hackathon.siim.org/fhir/

Registration for an api key for SIIM members [9] can be done at:

- https://siim.org/page/2017_hackathon_agree

A variety of free and public servers are also available to software developers for testing.

Toronto’s University Health Network (UHN) hosts a convenient public set of FHIR servers prepopulated with a large amount of test data and readily accessible through a user-friendly browser interface [10].

**Fig. 1:**
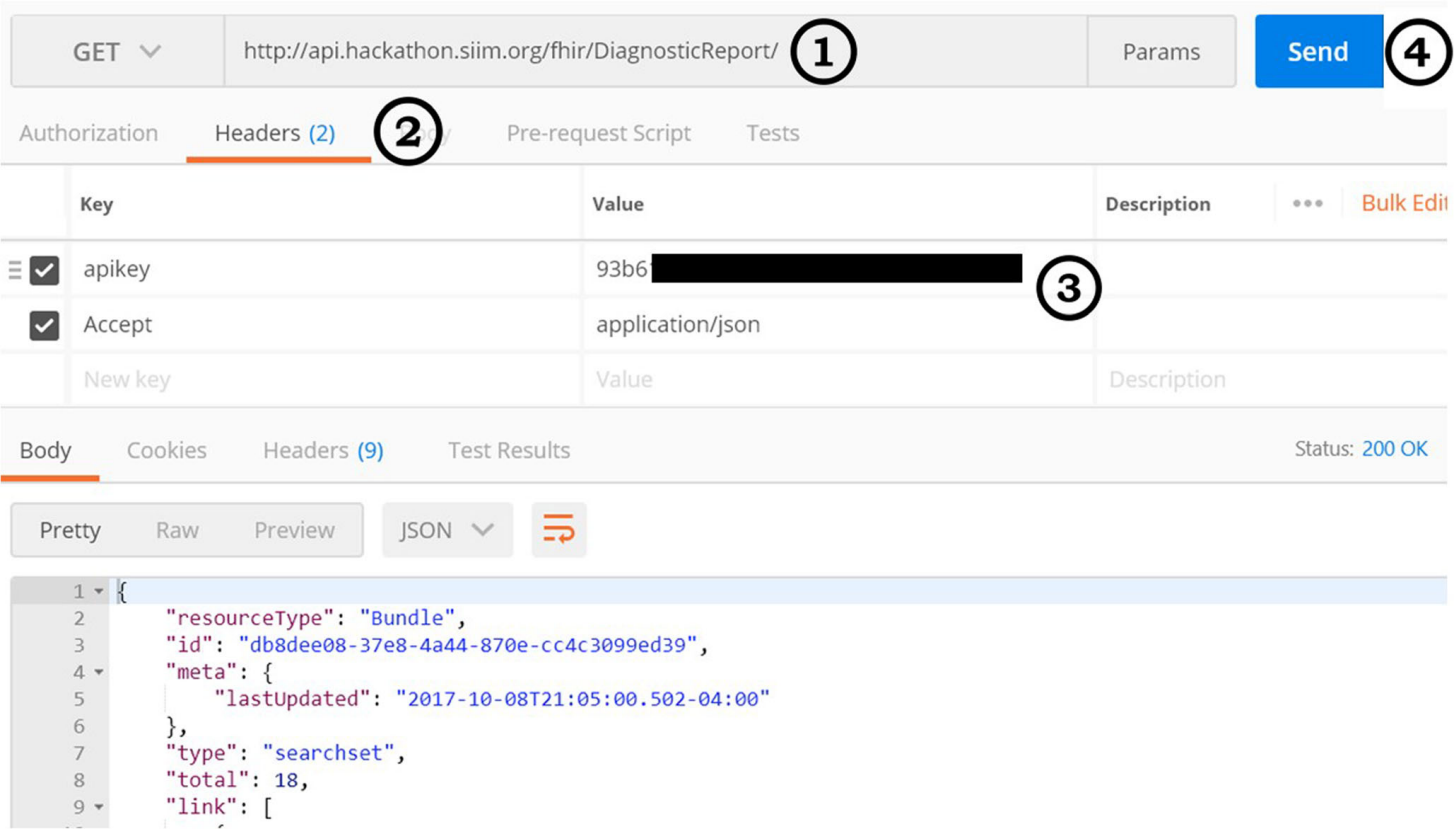
A screenshot of the Postman application configured to use the SIIM Hackathon FHIR server. (1) The URL that is being queried. (2) The Headers tab to specify which headers are included in requests to the server. (3) The SIIM FHIR server requires the header “apikey” with a valid apikey value to be sent in all requests to the server, which can be obtained by registering for the SIIM Hackathon. The “Accept” header can also be used to specify the return language of the results. (4) Pressing the send button will query the server and display the returned data

Several of these servers can be accessed with simple URL calls through the browser, allowing testing of queries and analysis of responses returned. For querying the SIIM FHIR server as recommended in the hackathon, a free tool called Postman (available https://www.getpostman.com/) permits users to query a FHIR server in an easy-to-use interface and view the results without writing code. Figure 1 shows how to configure Postman to use the SIIM Hackathon FHIR server, noting how to include the required apikey in the application calls to the server.

In practice, an application would make the API calls to the FHIR server to obtain data and display it in the software’s user interface. Note that many of these FHIR servers are in variable stages of development and may not support all features at this time, and the servers and standards themselves are subject to active revision which may have changed from this writing. It is likely that many of the queries in this article will require modification with ongoing changes to FHIR and updates to the SIIM FHIR server; however, the fundamental principles and design of the queries will be similar.

### FHIR Structure

The fundamental building block of FHIR is called a “resource”—or the type of information of interest in the EMR one is seeking to obtain. For example, a specific patient can be queried by accessing the “Patient” resource. A particular physician can be queried through the “Practitioner” resource. The “DiagnosticOrder” resource contains the orders placed by clinicians for imaging studies and can be used to generate worklists, as described in the following section (note: this has been named “ProcedureRequest” in FHIR Release 3). Table 1 highlights the FHIR resources most relevant to medical imaging. The complete list of the FHIR resources is well-documented on the HL7 website [12]:

- https://www.hl7.org/fhir/resourcelist.html

**Table 1:** A list of FHIR resources that are most relevant to radiology with a short description of each and an example of a potential use in integrated radiology software

Resource	Description	Potential use in radiology software
AllergyIntolerance	A record of allergies and adverse reactions to substances	Checking allergic reaction to contrast material
Condition	A record of diagnoses, problems, and clinical conditions	Displaying a patient’s active medical diagnoses. This resource can also be used for machine discoverability of studies for artificial intelligence research [11]
DiagnosticOrder (ProcedureRequest in FHIR Release 3)	The orders and requests placed by clinicians for imaging or laboratory studies	Generating a worklist based on requested imaging studies
DiagnosticReport	The reports for laboratory, pathology, and imaging tests	Storing and retrieving radiology reports
ImagingStudy	A representation of a DICOM imaging study	Accessing DICOM information to allow for image retrieval from the appropriate DICOM server
Observation	A general location for lab results, vitals, and other patient measurements	Accessing relevant lab data such as last creatinine
Patient	The information about the patient receiving the health care service	Obtaining basic information such as date of birth and gender

Constructing a query is as simple as putting the name of the desired resource at the end of the server URL. For example, all DiagnosticOrders can be accessed with the URL:

- http://api.hackathon.siim.org/fhir/DiagnosticOrder 1

**Fig. 2:**
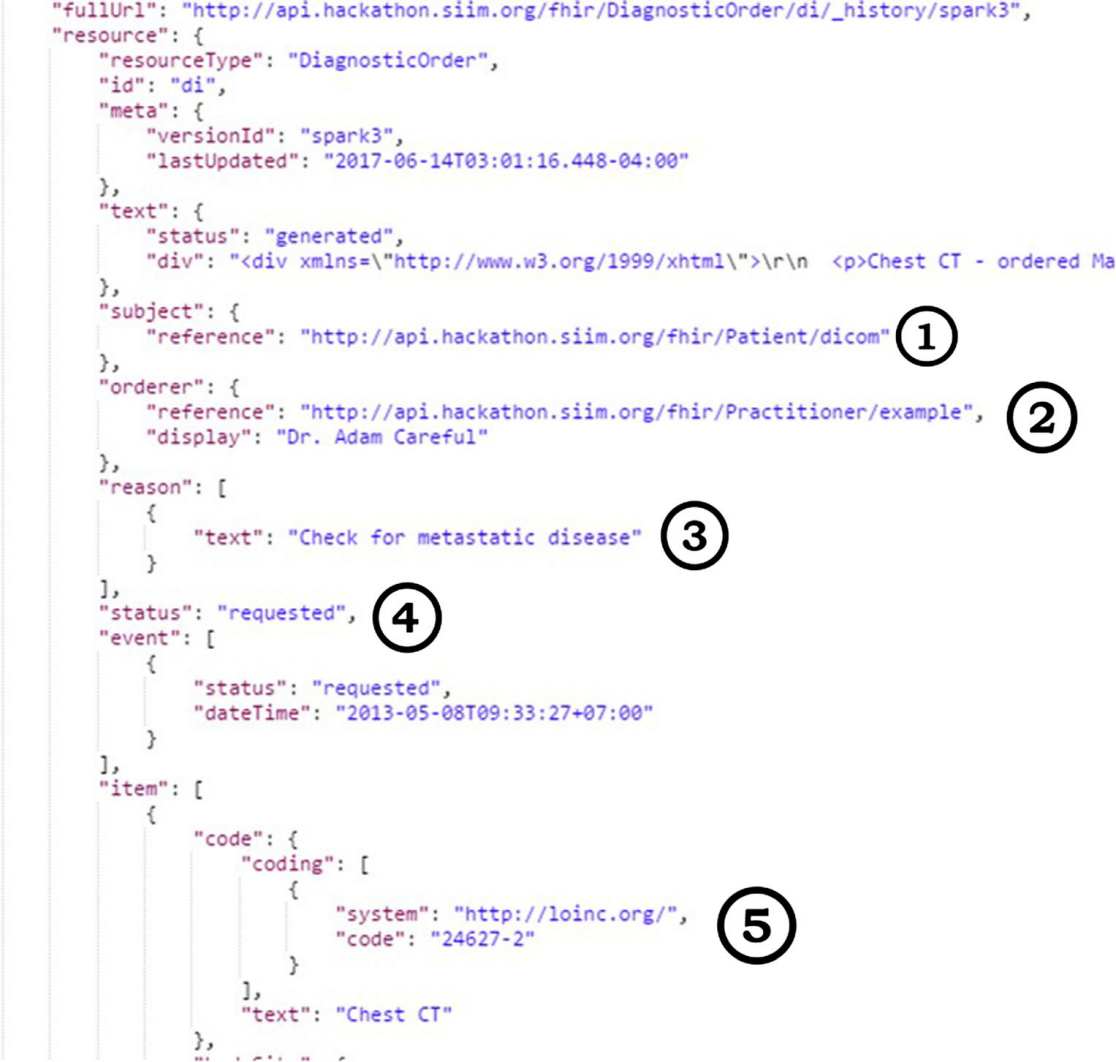
Sample results returned for querying/DiagnosticOrder as well as the type of data stored in the DiagnosticOrder resource such as (1) the patient on whom the study is ordered, (2) the ordering clinician, (3) the documented indication, (4) the status of the request, and (5) the coding that specifies the imaging type

Querying the URL will display the results of all DiagnosticOrders—regardless of status or type of study—in a standardized format called JavaScript Object Notation (JSON) which is a human-readable text that is commonly used to pass data in development environments and can be processed in a variety of software tools. The results of this query include data for a large number of DiagnosticOrders as illustrated in Fig. 2. Each individual DiagnosticOrder contains additional elements that include information such as the status of the order, the physician who placed the order, and the specific type of order. These attributes give an indication of how the resource can be queried.

### Constructing Queries: Generating a Worklist

The power of FHIR comes from the ability to construct queries with parameters to specify a search. For example, the DiagnosticOrder resource contains a “status” parameter which indicates whether an order has been requested, completed, or canceled. All pending orders that have been requested but not yet completed can be queried by including the “status” parameter in the query as follows:

http://api.hackathon.siim.org/fhir/DiagnosticOrder?status=requested

In the setting of radiology, these parameters can be used to generate worklists and protocol lists in conjunction with parameters that specify the imaging modality or body site.

The DiagnosticOrder resource can be queried by the parameter “code,” where “code” is a numeric representation of the type of order, such as a radiograph of the abdomen or a CT scan of the chest. As displayed in Fig. 2, the code for a CT chest is “24627-2”, noting that the system of coding refers to the Logical Observation Identifiers Names and Codes (LOINC) database [13]. Querying for all pending chest CTs would then be as follows:

http://api.hackathon.siim.org/fhir/DiagnosticOrder?status=requested&code=24627-2Queries like this can be used to generate radiology worklists and can also serve to dynamically compute metrics such as the number of pending studies.

### The Patient-Centered Approach: Protocoling and Reading Studies

The patient-centered approach that is made possible through FHIR comes from the ability to obtain clinically relevant patient information and integrate it directly with the medical imaging software that is used to protocol or read studies. Clinical information can then be actively used within the radiology workflow rather than being locked away in the EMR.

The following queries demonstrate how to first access information related to the patient and subsequently use it to query for information relevant to protocoling and reading studies.

Most resources contain an element called “subject” or “patient” and this is a link to access the resource of the patient, typically of the form “Patient/id” such as:

Patient/123

“123” here refers to the patient’s identifier in the FHIR server. Accessing the Patient resource can be done as:

- http://api.hackathon.siim.org/fhir/Patient/123

This query will provide basic information such as the patient’s gender and date of birth.

The patient identifier can be used in conjunction with other FHIR resources to search for relevant patient data in the EMR. For example, when protocoling a study, the radiologist requires information related to the patient’s kidney function from a lab test called the creatinine in addition to information such as the patient’s allergies.

Searching for the patient’s creatinine with FHIR can be done with a single query as follows:

- /Observation?patient=123&code=2160-0

In this query, Observation is the resource that contains the results of laboratory data, 123 refers to the patient’s identifier, and 2160-0 is the LOINC code for creatinine.

Retrieving the patient’s allergies can similarly be performed with the patient identifier parameter on the AllergyIntolerance resource:

- /AllergyIntolerance?patient=123

This query would generate a list of the patient’s allergies. With FHIR, a single query can be used to search specifically for contrast allergies:

- /AllergyIntolerance?patient=123&code=407935004

where 407935004 is the Systematized Nomenclature of Medicine (SNOMED) code for contrast media.

When reading a study, a radiologist often utilizes information such as a history of the patient’s surgeries, laboratory values, and clinical notes to provide a more educated interpretation of a study.

Generating a list of a patient’s prior surgeries can be performed by querying the Procedure resource:

- /Procedure?patient=123

Laboratory values can also be easily integrated into the reading environment. For example, a radiologist reading a thyroid scan can have access to laboratory tests that indicate the patient’s thyroid function via:

- /Observation?patient=123&code=24348-5

where 24348-5 is the LOINC code for the thyroid-stimulating hormone panel.

FHIR also provides the potential of querying clinical notes. With FHIR, a single query could be used to access the last clinical assessment written by the ordering provider of the study. Each DiagnosticOrder resource contains a reference to a Practitioner resource who ordered the study in the format:

- /Practitioner/5144

where 5144 refers to the practitioner’s identifier on the FHIR server. The Practitioner resource contains useful information such as the clinician’s contact information and can be used as a parameter with other resources to query for assessments or notes. A query for the last clinical note written by the ordering provider of the study could be performed via:

- /ClinicalImpression?patient=123&assessor=5144

where ClinicalImpression is a resource in development for storing clinical notes.

## Discussion

### The 2016 SIIM Hackathon Implementation

**Fig. 3:**
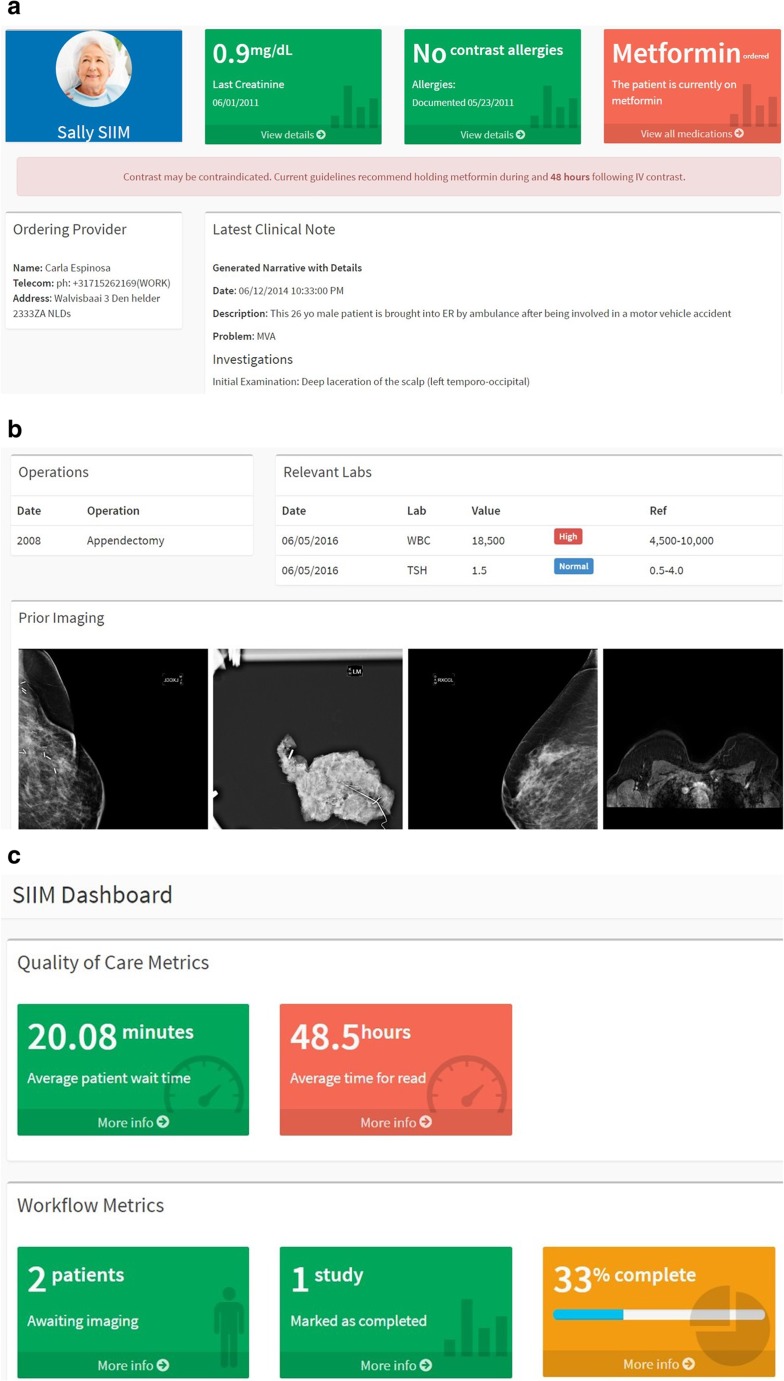
**a** A sample of a clinically integrated platform highlighting information relevant for the radiologist such as last creatinine and contrast allergies as well as providing the latest clinical note written by the ordering provider all pulled in real-time from the FHIR server. **b** Additional information such as previous surgeries and relevant lab work, as well as embedded DICOMWeb images included in a single platform. **c** A dashboard displaying quality metrics dynamically generated from FHIR and additional SIIM Workflow Initiative in Medicine (SWIM) API data

The annual SIIM Hackathon challenges users to develop software integrations using standards like FHIR to innovate new solutions to healthcare problems. Samples from the 2016 SIIM Hackathon are included in Fig. 3a–c and illustrate what a fully integrated system with FHIR and DICOMWeb could look like in the radiology workplace. Instead of having a worklist driven by imaging, at the forefront is the patient with the relevant clinical information logically organized (Fig. 3a, b). With API tools like DICOMWeb, medical images can be directly embedded into the clinical workflow (Fig. 3b) [14]. API access to the data in the FHIR server also offers real-time quality metrics **(**Fig. 3c**)**. All these features can be streamlined into a single platform which can unify the radiology workflow into a cohesive user experience driven by a patient-first clinical focus.

## Summary

FHIR is an exciting and promising new standard that is expected to drive great technologic innovation. As access to data is made easier, the development of new software tools and applications that will solve a variety of clinical challenges and streamline workflow will flourish. It has been shown that by eliminating barriers by making health data accessible, application development, innovation, and technologic growth is the natural result [15, 16]. This is particularly true with a standard like FHIR that is developer-friendly and up-to-date with current technology standards. In the field of radiology, FHIR can serve to integrate information previously separated across different software platforms and offer a more clinically relevant and patient-centered workflow.

## Footnotes

1 Note: the DiagnosticOrder resource is being replaced by a general ProcedureRequest resource in FHIR Release 3.0, which would require modification of the queries for up-to-date FHIR servers to http://api.hackathon.siim.org/fhir/ProcedureRequest.
